# Implementation of the electronic community health information system in rural East Shewa zone, Eastern Ethiopia: a CFIR-ERIC framework for facilitators, barriers and implementation strategies

**DOI:** 10.3389/fdgth.2025.1554995

**Published:** 2025-07-07

**Authors:** Serbesa Dereje Degaga, Solomon Shiferaw Yesuf, Girma Taye Aweke

**Affiliations:** School of Public Health, College of Health Sciences, Addis Ababa University, Addis Ababa, Ethiopia

**Keywords:** barriers, facilitators, eCHIS, maternal health services, CFIR, ERIC, Oromia, Ethiopia

## Abstract

**Background:**

The Electronic Community Health Information System (eCHIS) is a key initiative of Ethiopian government to digitize the Health Extension Program and enhance community health services. Its effective implementation requires identifying key barriers and facilitators to inform tailored strategies. This study employed the Consolidated Framework for Implementation Research (CFIR) to explore implementation determinants and used the Expert Recommendations for Implementing Change (ERIC) to develop context-specific strategies aimed at strengthening eCHIS adoption and sustainability.

**Methods:**

An exploratory case study design was employed in rural districts of East Shewa Zone, Eastern Ethiopia. Data were collected from November 5 to 25, 2024. A total of 24 face-to-face in-depth interviews were carried out with key informants across multiple sites. All interviews were audio-recorded, transcribed verbatim, and subsequently translated into English for analysis. Data were coded using Open code 4.03 software and thematically analyzed. Findings were interpreted using the CFIR–ERIC framework.

**Results:**

Within the Intervention Characteristics of CFIR domain, barriers included perceptions of eCHIS as an externally imposed system and the lack of interoperability with existing systems. Conversely, the comprehensive design of eCHIS, including integrated job aids, was recognized as a facilitating factor. In the Outer Setting domain, weak community engagement and the absence of clear implementation guidelines were cited as major barriers. For the Inner Setting domain, data synchronization challenges were highlighted as barriers, while the provision of tablets and accessories was identified as a facilitator. Under the Characteristics of Individuals domain, strong self-efficacy and a belief in eCHIS's positive impact on service delivery were noted as facilitators. Finally, in the Implementation Process domain, the lack of planning and leadership engagement was perceived as a barrier, whereas the availability of technical support and feedback mechanisms emerged as critical facilitators. Seven strategies to enhance implementation were developed such as Strengthening leadership engagement, developing clear guideline, fostering community engagement, creating system interoperability, establishing robust feedback systems, integrating health worker incentives program and enhancing technical support.

**Conclusion:**

The study offers key insights into the barriers, facilitators, and strategies of eCHIS implementation. Applying these strategies could strengthen implementation, enhance adoption, and promote long-term sustainability.

## 1 Introduction

Over the past two decades, mobile health (m-Health) has been recognized as a groundbreaking and transformative approach in the global digital health landscape. It has significantly contributed to the modernization of healthcare delivery. It leveraged mobile technology to improve access, efficiency, and quality of care (1). The Global observatory for eHealth defines mHealth as the practice of medicine and public health supported by mobile devices such as mobile phones, patient monitoring systems, personal digital assistants (PDAs), and other wireless technologies. Considering the shortage of skilled human resources and increasing accessibility in remote areas, mobile phones are increasingly acknowledged as a valuable tool for facilitating effective and sustainable communication within the health sector (2). Mobile technology plays a major role in sexual and reproductive health care and is seen as a key area in achieving the global targets for women's and children's health (3). The ministry of health introduced mobile-based application, the electronic community health information system (eCHIS) in 2018 as part of its information revolution agenda (4).

eCHIS aims to transform Ethiopia's health extension program (HEP) from paper-based system to a technology-driven platform to enhance health care delivery system (5). The HEP is Ethiopia's flagship health initiative that was initiated in 2003 to ensure universal access to primary health care at periphery level (6). eCHIS comprises four key modules: The Digital Family Folder; Reproductive, Maternal, Newborn, Child Health and Nutrition (RMNCHN); Disease Prevention and Control (DPC) and Logistics Supply and Management (5).

It offers numerous advantages by facilitating improved healthcare delivery and outcomes particularly in resource-constrained community settings (7). Key benefits include the provision of electronic decision support, referral processes, and patient tracking (8); The system also reduces redundancy in data recording and reporting while enhancing communication among healthcare providers (9, 10). Furthermore, eCHIS contributes to improved data accuracy and integrity, thereby strengthening evidence based decision-making (11). Specifically, the eCHIS-RMNCHN enables healthcare providers to comprehensively assess maternal health status and make informed decisions regarding the provision of care. It incorporates detailed counseling guidelines on obstetric danger signs and complications during pregnancy, childbirth, and the postpartum period. It also facilitates the continuum of care through longitudinal client tracking and integrated electronic referral systems. Furthermore, the module allows midwives at health centers to offer feedback on referrals made by Health Extension Workers (HEWs) within their catchment areas (12).

Despite the substantial benefits of digitizing health information systems, global evidence indicates that mHealth initiatives in low- and middle-income countries (LMICs) continue to face persistent implementation challenges. These include limited network connectivity, low levels of digital literacy, frequent power outages, difficulties in device maintenance, inadequate infrastructure, concerns regarding trust in the technology, and insufficient incentives for frontline health workers (13, 14).

In sub-Saharan Africa, similar studies highlighted that mhealth implementation faces momentous challenges due to limited access to network and electricity (15, 16); limited political support and user acceptance (17); interoperability issues between systems (9); resistance to adopting digital tools (18); education level of health workers (15); Privacy and confidentiality concerns (19, 20); limited access to cell phones (16); lack of trust among stakeholders, particularly when integrating disparate systems owned by different parties (17, 19).

In Ethiopia, the utilization of eCHIS for maternal health services also remains suboptimal with significant regional variations in implementation. Among the 17,903 health posts nationwide, 7,808(46%) have adopted eCHIS since 2018. But, only 1,912 (24.5%) offer ANC services, and 795 (10.2%) provide PNC services using this platform (21). While acceptance rates among HEWs are high (94.4%–97.4%), utilization lags at 50% (22). Inadequate infrastructure, poor supervision, dual reporting systems, heavy workload, limited connectivity, staff turnover, competing priorities, lack of incentives, weak ownership, low digital literacy, and limited peer support and self-efficacy hindered eCHIS utilization (23, 24).

Although existing studies on eCHIS have predominantly explored its barriers and facilitators, there remains a critical gap in generating tailored, evidence-informed strategies to guide its effective implementation, adoption, and long-term sustainability. The Consolidated Framework for Implementation Research (CFIR) posits that the success or failure of an implementation is determined by various factors, including the characteristics of the intervention itself, the external context or outer setting, the internal organizational context or inner setting, and the attributes of individuals involved in the process (25). By integrating CFIR with the Expert Recommendations for Implementing Change (ERIC), tailored strategies can be developed to address barriers and leverage facilitators at various levels. This combined approach enables systematic identification of challenges and application of targeted solutions for successful implementation initiatives (26).

Thus, this study aimed to explore the barriers and facilitators influencing the utilization of the eCHIS-RMNCHN module using the Consolidated Framework for Implementation Research (CFIR). It further sought to develop context-specific implementation strategies by linking these findings to the Expert Recommendations for Implementing Change (ERIC) to support effective implementation, foster adoption, and ensure the long-term sustainability of the system.

## 2 Materials and methods

### 2.1 Study design, setting and period

The study used an exploratory case study design. This design is a valuable approach to explore the real-world application of interventions, programs, or policies in their natural settings. It allows researchers to investigate the processes, challenges, and contextual factors that influence the implementation and outcomes of specific interventions (27). We employed the design to identify barriers, facilitators, and implementation strategies for evidence-based implementation of eCHIS-RMNCHN module. Data were collected from five primary health care units (PHCUs) within East Shewa Zone of Eastern Ethiopia from November 5 to 25, 2024.

### 2.2 Participants and sampling

We utilized a purposive sampling technique to select Health Extension Workers (HEWs), midwives, HEW supervisors, Primary Health Care Unit (PHCU) directors, health information technicians (HITs), and woreda health officials (district health managers).Participants were purposively selected based on their extensive experience and their capacity to provide rich insights into the barriers and facilitators of eCHIS implementation. Eligible participants were health workers aged 18 years or older who voluntarily participated and provided written informed consent. A total of 24 key informants representing diverse roles were involved to obtain in-depth insights. The sample size was determined based on the principle of data saturation, whereby interviews were concluded once no new or relevant information emerged from participants. Respondents were selected from Primary Health Care Units (PHCUs) and district health offices where the eCHIS-RMNCHN module has been implemented for at least one year.

### 2.3 Data collection tools

The semi-structured interview guide was constructed based on the Consolidated Framework for Implementation Research (CFIR). This framework was adopted because it is a comprehensive and helps to assess a wide range of factors (25). Open-ended questions covering all five domains of the CFIR framework (intervention characteristics, inner setting, outer setting, process, and characteristics of individuals) were adapted from the CFIR online interview guide tool to explore eCHIS implementation barriers and facilitators (28). The interview guides were developed in local languages (Afaan Oromoo and Amharic), pre-tested, and subsequently used to collect data across all levels.

### 2.4 Data collection procedures

The data were gathered by the principal investigator, a specialist in public health with extensive experience in qualitative data collection. Total of 24 participants were involved in the interview. Interviews were conducted in-person in a private setting of their choice. All participants were encouraged to express their views freely in response to the interview questions. Interviews continued until data redundancy was reached, and no additional new or relevant information emerged. Respondents' responses were recorded using a tape recorder and additional notes were documented throughout the interview process. Follow-up questions were employed to facilitate an in-depth exploration of the topics discussed. To ensure comprehensive reporting of all elements of the qualitative research, the Consolidated Criteria for Reporting Qualitative Research (COREQ) was employed as a guiding checklist (29).

### 2.5 Ethical considerations

Ethical approval for this study was obtained from the Institutional Review Board of the College of Health Sciences, Addis Ababa University (Protocol No. 059/24/SPH, dated 01 November 2024). Prior to data collection, official permission was also secured from the respective local administrative authorities in the study areas. All participants were provided with detailed information about the study's objectives, procedures, potential risks and benefits, and their rights as participants. Written informed consent was obtained from each participant before participation. Emphasis was placed on the voluntary nature of participation, including the right to withdraw from the interview at any stage without the need to provide a reason. To ensure confidentiality, all interviews were anonymized through the study. Each transcript was assigned a unique identifier to prevent the disclosure of personal information. Audio recordings and transcripts were securely stored on a password-protected computer, accessible only to the research team.

### 2.6 Data process and analysis

The audio recordings were transcribed verbatim in the original language of the interviews and subsequently translated into English. The data were then transferred to Open Code 4.03 software for coding and analysis. An iterative deductive approach was employed for coding. The coding was guided by pre-existing Consolidated Framework for Implementation Research (CFIR) domains and constructs (25). Thematic analysis was conducted. To enhance the rigor and credibility of the analysis, several measures were taken such as repeated readings of the transcripts to ensure consistent coding, the use of a clearly defined coding framework based on CFIR constructs, and regular consultations among research team to review the coding structure and interpretation of the findings. Similar codes were grouped and categorized into two overarching themes (barriers and facilitators). Key quotes that exemplified these themes were selected and presented. Following the identification of implementation barriers and facilitators, the themes were rigorously reviewed and aligned with existing evidence. Based on considerations of importance, feasibility, and sustainability, seven strategies were developed through a series of discussions. Finally, the Expert Recommendations for Implementing Change (ERIC) framework was utilized to identify and link the mechanisms of action for each implementation strategy (26) (See Table 2).

## 3 Results

### 3.1 Socio-demographic characteristics of participants

In-depth interviews (IDIs) were conducted with 24 health workers, comprising eleven males and thirteen females, with ages ranging from 23 to 47 years. The participants had professional experience ranging from two to twenty-three years. The participants consisted of three woreda health officials, four Health Information Technicians (HITs), three Primary Health Care Unit (PHCU) directors, five midwives, three Health Extension Program coordinators, and six Health Extension Workers (HEWs).

### 3.2 Barriers and facilitators to implementing eCHIS program

**Figure 1:**
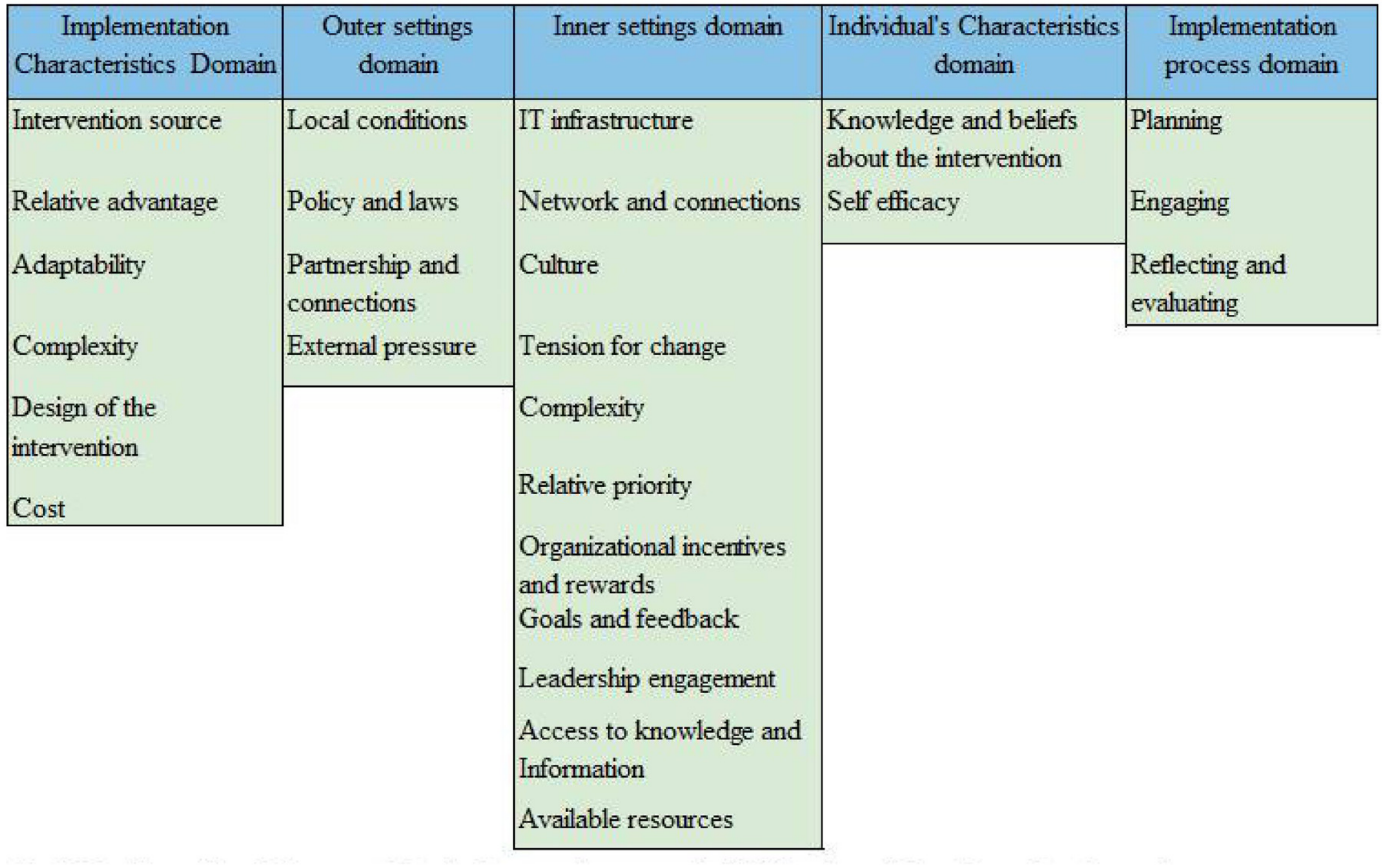
The consolidated framework for implementation research (CFIR).

The study found that the key facilitators of eCHIS implementation were alignment of eCHIS with national health priorities, belief that it improves service delivery, its comprehensive design including the integrated job aids, its user-friendliness and availability of tablets and accessories. However, its full potential was hindered by unreliable internet connectivity, underdeveloped reporting functionalities, unclear implementation guidelines, lack of system interoperability, and limited leadership engagement. To address these challenges, seven implementation strategies were developed by integrating the Consolidated Framework for Implementation Research (CFIR) with the Expert Recommendations for Implementing Change (ERIC). These include strengthening leadership engagement, developing clear guidelines, enhancing training and support, fostering community ownership, improving interoperability and reporting, establishing feedback systems, and integrating eCHIS into health worker incentives, aiming to optimize its use and impact on health service delivery. The key facilitators and barriers to eCHIS implementation were discussed thematically based on the domains and constructs adapted from the Consolidated Framework for Implementation Research (25) (See Figure 1).

#### 3.2.1 Theme 1: Intervention characteristics domain

##### 3.2.1.1 Intervention sources

Most respondents recognized that the eCHIS was introduced and implemented as part of the Ministry of Health's Information Revolution agenda, with technical and logistical support from different development partners. Partners' collaboration and alignment with a national governmental initiative has significantly contributed to the program's credibility and fostered trust among stakeholders to strive for the strategic vision of the Ministry. However, a small number of health workers believe that eCHIS was introduced and implemented by an external organization, such as JSI L10K. “*eCHIS was implemented by JSI L10K and is a good system designed to improve service delivery and enhance data management and quality at the community level.”* (P8, Female).

##### 3.2.1.2 Relative advantage

The majority of respondents acknowledged that the utilization of eCHIS enhanced their service and data quality. *As an HEW, I found eCHIS highly beneficial for healthcare management. It streamlines data compilation, enhances data security, prevents inaccuracies, and simplifies kebele profile updates and appointment tracking with its color-coding system*. (P3, Female). eCHIS additionally contributes to the integrity of data by mitigating manipulation. “Since eCHIS archives all recorded data in a fixed, unalterable format*, it prevents the possibility of data fabrication. This feature has led some health extension workers to prefer paper-based systems, which allow for more flexible manipulation of data."* (P12, Male) All health officials and health workers witnessed the benefits of eCHIS in provision of timely and quality health care services. *“I can say that eCHIS is a means by which we can reduce maternal and child mortality for it retains women in the continuum of care”. (P4, Female).*

##### 3.2.1.3 Adaptability

Most midwives and health extension workers reported a significant increase in familiarity and engagement with the eCHIS system over time. “*Since its inception, the eCHIS platform has undergone numerous revisions, with each version showing progressive enhancements based on the feedback provided during implementation. The system is easy for customization to meet our specific operational needs”*. (P5, Male).

##### 3.2.1.4 Complexity

Nearly all respondents indicated that they did not encounter challenges in using eCHIS for delivering healthcare services. One respondent, (P13, Famale), explained, “*The utilization of eCHIS is straightforward, with clear steps that guide the user through the process and the terminologies used in the system are familiar”* Another respondent (P1, female) added, “ *The procedures of eCHIS are simpler compared to the more cumbersome paper-based formats we previously used.*”

##### 3.2.1.5 Design

Respondents expressed a sense of ease and confidence in utilizing the eCHIS system. One respondent highlighted its comprehensiveness, noting, “*eCHIS is comprehensive; it provides various job aids in the form of images and audio, which support me in delivering quality services to clients. These tools serve as reminders for information I may overlook during counseling sessions”*. (P6, Female). However, other respondents indicated that the reporting functionality of eCHIS is not yet fully developed for efficient data exchange and comprehensive reporting purposes. “*Although eCHIS includes a reporting feature, we continue to rely on manual methods for reporting”*. (P9, Female). “*Ensuring interoperability between the eCHIS and DHIS2 systems is essential for establishing an effective reporting system”*. (P10, Male).

##### 3.2.1.6 Cost

Most respondents explained that they have no awareness regarding the costs associated with the implementation of eCHIS. They expressed that their focus is only on successful execution of the program to achieve the Ministry of Health's objective of digitizing health service delivery*. “I am not aware of the specific costs involved in the implementation of eCHIS. But, I know that the Ministry of Health in collaboration with development partners has provided essential resources such as tablets, SIM cards, airtime, and power banks, to facilitate the implementation of eCHIS”. (P16, Male).*

#### 3.2.2 Theme 2: Outer setting domain

##### 3.2.2.1 Local conditions

Respondents witnessed that decentralization of government structures made in Oromia region has created a favorable environment for the effective utilization of eCHIS. “*One of the challenges in implementing the Health Extension Program, including eCHIS, has been the fact that many health extension workers do not reside within the communities they serve. Instead they opt to live in towns far from those communities. The decentralization enhanced the delivery of healthcare services and improved oversight of both the Health Extension Program and health extension workers.” (P2, Male).*

##### 3.2.2.2 Policy and laws

The lack of clear guidelines for eCHIS implementation has been identified as a significant challenge. Respondents reported difficulties in determining which population data source to use for performance calculations, planning, and resource allocation whether to rely on the population data provided by the FMOH or the data collected through eCHIS at the community level. “*….HEWs believes they should be evaluated based on actual population data collected on the ground* via *eCHIS. But, their performance is calculated using conversion factors based on the population data that was enumerated 15 years age and this demoralized them due to perceived underperformance”.* (P17, Male).

Another respondent expressed his thoughts by stating, *“For instance, in one kebele within our woreda, the maternal and child health performance rate, based on the population data provided by the FMOH, is calculated to be no more than 40%. However, this figure could rise to 80% or 90% if we use the population data registered in eCHIS”. (P2, Male).*

##### 3.2.2.3 Partnerships & connections

There is good communication between the Federal Ministry of Health (FMOH) and Ethio-telecom to address internet connectivity challenges to ensure the implementation of the electronic Community Health Information System (eCHIS). “ *Although it is not entirely sufficient, 100MB of data is provided monthly with the support of the Federal Ministry of Health (FMOH) and development partners to facilitate data synchronization and referral activities”* (P9, Female).

However, The benefits of the electronic Community Health Information System (eCHIS) in enhancing the provision of quality maternal health care were not effectively communicated as a new initiative to the community community-level structures, including the newly kebele level established structure, Women Development groups and the One-to-five networks. “*Only the midwives, HITs and we (HEWs) are actively engaged in and concerned about the implementation of eCHIS”. (P18, Female).*

##### 3.2.2.4 External pressure

The study indicated that the absence of peer support following training sessions, along with limited engagement from key program stakeholders, such as Maternal and Child Health staff at the woreda health office level, has impeded the successful implementation of the electronic Community Health Information System (eCHIS). Additionally, the absence of strong advocacy efforts, coupled with the limited use of mass media for promotion, may hinder the effective implementation of the electronic Community Health Information System (eCHIS). “*Leaders and mass media have given minimal attention to the electronic community health information system (eCHIS) compared to other initiatives like community-based health insurance despite eCHIS being a major initiative”. [P20, Male].*

#### 3.2.3 Theme 3: Inner setting domain

##### 3.2.3.1 Information technology infrastructure

The procurement and distribution of tablets, SIM cards, power banks, and the provision of monthly airtime considerably facilitated the implementation of the electronic Community Health Information System (eCHIS). However, weak internet connectivity posed substantial challenges to its effectiveness. One of the respondents (P15, Female) shared her thoughts, stating “*Despite airtime top-ups, there are instances where the system becomes unresponsive during the recording of client information”.* Another participant (P14, Female) described the challenges as “*I have experienced internet outages lasting over a week, preventing data synchronization and disrupting the referral system, which hinders my ability to refer clients to midwives”.*

##### 3.2.3.2 Networks and communications

While not fully matured, the dashboard feature in the eCHIS application has supported communication and enabled implementers to provide feedback on implementation progress. Many respondents also noted that the creation of a Telegram group has enhanced their communication. One respondent (P11, Male) stated, “I *use the dashboard to track the performance of each PHCU and provide feedback through the telegram group created for communication”*.

##### 3.2.3.3 Culture

Most of the respondents highlighted that inadequate team collaboration and communication were key obstacles to the successful implementation of the eCHIS system. A respondent (P19, Female) conveyed her perspective, remarking, “*When eCHIS was introduced, we weren*’*t really emphasized to work with other staffs. Everyone seemed to be doing their own task”.* Additionally, a prevailing resistance to change, with staff maintaining a preference for traditional paper-based reporting methods, further compounded these challenges. A respondent (P13, Male) articulated his feelings, noting “*Some healthcare workers resist using eCHIS because it doesn*’*t allow fabrication of data, which they could do with the paper-based system*”.

##### 3.2.3.4 Tension for change

Respondents consistently emphasized that the implementation of the eCHIS was seen as a critical driver for improving healthcare service delivery. A respondent (P21, Female) expressed her sentiments, stating, “*Before eCHIS, we were using paper records, and it was hard to keep track of our clients. But, now, it is much easier to follow up our clients across their care journey because eCHIS shows clients’ next appointment with color coding*” Many respondents also highlighted that eCHIS enhanced the quality of services by improving counseling processes and facilitating effective client tracking for continuity of care. “*eCHIS really guides us during counseling sessions. It ensures that we are giving the right information to the clients, which enhances the overall quality of the counseling.” (P4, Famale).*

##### 3.2.3.5 Compatibility

All most all respondents mentioned that the eCHIS is digitalized form of Health Extension Program (HEP). They expressed that eCHIS not only aligns well with the existing workflows but also significantly strengthens the communication and data-sharing processes between health posts and health centers. A respondent (P2, Male) stated, “*Digitalizing HEP via eCHIS has made things smoother. It is compatible with how we have been working for years and the tools are familiar”.* Another respondent added, “*eCHIS is not only a digital version of HEP, but it also improves how quickly we can get information from health posts to the health centers” (P13, Male).*

##### 3.2.3.6 Relative priority

Many respondents expressed concerns that eCHIS implementation is not considered a high-priority initiative in their setting. Instead, they feel that Community-Based Health Insurance (CBHI) takes precedence due to its direct impact on healthcare access and financial protection for communities. A respondent (P17, Male) expressed his feelings, “Community Based Health Insurance (*CBHI) is one the government initiative and our main focus is on expanding the program*.” In a similar vein, another (P8, Female) respondent remarked*, “Although eCHIS could be beneficial, our efforts are being directed towards the Community-Based Health Insurance initiative. eCHIS takes a back seat in terms of priority*.”

##### 3.2.3.7 Organizational incentives and rewards

Many respondents explained that their health institution has not established any formal recognition or reward systems specifically tied to the implementation of the eCHIS initiative. They expressed significant concerns regarding the absence of organizational incentives, which could be either financial or non-financial (such as public recognition, certificates of appreciation, or opportunities for professional development). The lack of these incentives has led to a widespread feeling of demotivation among staff. “*It is disappointing that despite the extra effort required to adopt eCHIS, there are no special rewards and even recognition for those of us who have been early adopters. It's demotivating when you don*’*t feel valued for your effort”* (P3, Female).

##### 3.2.3.8 Goals & feedback

Respondents reported that no clear and measurable targets were communicated in relation to the implementation of the eCHIS-RMNCH module. One of the respondents (P9, Female) expressed her feelings, “*We know that RMNCHN module aims to improve continuity of care but there was no clear target set for us. We don*’*t know how much we should reduce dropout rates or what completion rates we’re aiming for”* Nevertheless, respondents recognized the utility of performance feedback provided through dashboards, even though the updates are infrequent. “*There is a time when we received feedback from upper which was generated from dashboard. Even though the feedback doesn*’*t come often, it helps us see where we are”.* (P14, Female).

##### 3.2.3.9 Leadership engagement

Respondents expressed that eCHIS implementation has not received the necessary attention and support from the newly established kebele leadership. They indicated that they expect the leaders to provide the same level of involvement and attention to eCHIS as they do with other initiatives such as the Community-Based Health Insurance (CBHI) program. *Since the new kebele structure was established, the leadership hasn't engaged with eCHIS. “The leaders haven't prioritized it like CBHI, and without their engagement, it's difficult to push the implementation forward”.* (P21, Female).

##### 3.2.3.10 Access to knowledge & information

Respondents expressed concerns about challenges in obtaining information regarding the eCHIS (Electronic Community Health Information System) intervention from program owners, particularly Maternal and Child Health (MCH) staff, as they are not fully informed about the utilization of eCHIS*.* One of the respondents, (P19, Female) shared her emotions; *“I haven*’*t got clear guidance from the MCH staff regarding eCHIS because they don*’*t seem to be fully aware of its functionality”.* However, respondents acknowledged that they receive the necessary information and support from Health Information Technicians (HITs) when needed. “*Whenever we face challenges with eCHIS, we can always count on the HITs. They are our main resource whenever we need assistance with implementation issues”.* (P9, Female).

##### 3.2.3.11 Available resources

Respondents positively acknowledged that the Ministry of Health has provided critical resources for the implementation of the eCHIS (Electronic Community Health Information System) intervention, including tablets, SIM cards, power banks, and airtime refills. These resources have been instrumental in ensuring that health workers can access the system in the field. “*We are grateful that we’ve received tablets and power banks, which make it easier to use eCHIS during fieldwork. Having SIM cards and airtime refills has really made a difference in ensuring smooth communication and synchronization”. (P18, Female).* However, respondents also expressed concerns regarding the no budget allocated for important activities like review meetings, supportive supervision, and mentorship, which are essential for the successful and sustainable implementation of eCHIS. “*We have what we need in terms of eCHIS equipment, but the budget for review meetings and follow-up is lacking. This limits our ability to monitor on our progress and make necessary improvements to the eCHIS implementation” (P2, Male).*

#### 3.2.4 Theme 4: Characteristics of individuals domain

##### 3.2.4.1 Knowledge & beliefs about the intervention

Almost all respondents believe that eCHIS is very effective in streamlining data compilation, reducing the risk of false reporting, ensuring data security, and keeping kebele profiles updated. One of the respondents (P9, Female) shared her sentiments, “*eCHIS is very helpful for compiling data and ensuring that the report we submit is accurate. It reduces the risk of false reporting and helps maintain data security”.* They also explained that the system's built-in appointment tracking feature, with color-coded reminders, has been particularly useful for managing patient follow-ups, and the electronic referral linkage. It strengthened the communication between the health extension workers and midwives. *A*nother respondent (P15, Female) shared her feelings, “*The feature that uses color codes to track appointment dates is very interesting. It helps us follow up with patients more effectively, ensuring that no one misses their appointments”.*

##### 3.2.4.2 Self-efficacy

Respondents expressed strong confidence in their ability to implement the eCHIS (Electronic Community Health Information System) intervention. They described that they received comprehensive training, the user-friendly nature of the system, and the provision of essential resources such as tablets, SIM cards, power banks, and refilled airtime as key factors that boost their self-efficacy. While network issues sometimes pose challenges, respondents feel equipped and prepared to effectively utilize the system in their daily operations. A respondent (P1, Female) articulated her feelings as “*The system is straightforward to use, and the training was very helpful. Having the tablet and airtime recharges means we have everything we need keeping the system running. I feel capable of managing it without difficulty*”. Likewise, another respondent shared her feelings, *“I feel confident in using eCHIS because the training we received was thorough and the system itself is quite user-friendly”.*

#### 3.2.5 Theme 5: Implementation process domain

##### 3.2.5.1 Planning

Respondents expressed that there is a lack of a clear and structured plan for the implementation of the eCHIS (Electronic Community Health Information System) intervention. They explained that, aside from general encouragement to adopt the system, no specific targets or measurable objectives have been set, and eCHIS has not been formally integrated into the annual operational plan. *"There is no clear plan in place for the implementation of eCHIS. We have been encouraged to use the system, but there are no specific targets and objects that we are working toward”. (P13, Male).*

##### 3.2.5.2 Engaging

The majority of respondents reported receiving technical assistance including development partners. However, concerns were raised regarding the absence of a formally designated person to oversee the eCHIS implementation process. Respondents further noted that, while a new structure at the kebele level has been established with well-educated individuals, these new leaders are not yet fully informed about the eCHIS initiative and its implementation. One of the respondents (P2, Male) expressed his sentiments, “*Even though the new kebele structure with educated individuals presents a great opportunity, they are not well informed about the eCHIS system”.* Additionally, respondents emphasized the lack of engagement with women's development groups, which are crucial for early referral of pregnant women to healthcare services, ensuring continuity of care.

##### 3.2.5.3 Reflecting & evaluating

Respondents expressed appreciation for the establishment of a dedicated Telegram group for communication and idea-sharing related to the implementation of the eCHIS (Electronic Community Health Information System). They also valued the use of a dashboard that monitors the system's utilization, with Health Information Technicians (HIT) providing regular updates via the Telegram group. *“There is a dashboard that tracks how well eCHIS is being used, and the HIT does a good job of generating reports from the dashboard and posting them in our telegram group”.* (P17, Male) However, concerns were raised regarding the absence of clearly defined evaluation criteria assessing progress toward achieving the continuum of care in maternal health. Respondents noted that, beyond tracking system usage, no specific indicators or measurable targets have been set to evaluate the overall performance of the eCHIS implementation. One of the respondents (*P5, Male*) shared his sentiments, “*There are no specific evaluation criteria to measure our progress towards achieving the continuum of care. We are monitoring utilization status, but we don*’*t have indicators to access whether we are actually improving services”)* (Table 1).

**Table 1:** Descriptions of applied CFIR domains, constructs, and identified emergent themes (barriers and facilitators) (*n* = 24).

CFIR domain	Constructs	Themes (barriers)	Themes (facilitators)
Intervention characteristics	Intervention source	• eCHIS is perceived as externally imposed	• Aligned with national priorities, improving credibility and trust.
	Relative advantage	• Preference for paper-based for data manipulation.	• Improves service data quality and information use
	Adaptability	• Lack of full reporting functionality and interoperability with DHIS2	• Customizable and user-friendly
	Complexity		• Easy to use, familiar terminologies
	Design		• Comprehensive design; includes job aids and decision-support tools.
Outer setting	Local conditions	• Lack of health workers residing in the communities	• Decentralization aids adoption
	Policy & laws	• Lack of clear guidelines on using population data	
	Partnerships & connections	• Weak community engagement.	• The start of partnership with Ethio-telecom to improve internet connectivity
	External pressure	• Absence of peer support, advocacy, and mass media promotion	
Inner setting	Information technology infrastructure	• Weak internet connectivity • Difficulty in data synchronization and referrals.	• Provision of essential hardware and resources (tablets, SIM cards, power banks) facilitated implementation.
	Networks & communications		• Availability telegram platforms
	Culture	• Cultural resistance to change, especially due to preference for paper-based systems.	• Perception of improvements in service delivery and client tracking
	Tension for change		• Compatible with the existing Health Extension Program (HEP)
Individuals' characteristics	Knowledge & beliefs about the intervention		• Belief that eCHIS improves service delivery and data management.
	Self-efficacy		• Strong self-efficacy and knowledge in using eCHIS
Implementation process	Planning	• Lack of clear planning and measurable objectives for eCHIS implementation.	• Availability of technical assistance
	Engaging	• Lack of leadership engagement at the kebele level.	
	Reflecting & evaluating	• Absence of evaluation criteria and measurable targets	• Availability of dashboards and feedback mechanisms

### 3.3 Potential implementation strategies for eCHIS program

#### 3.3.1 Strengthen leadership engagement and advocacy

This strategy focuses on addressing the lack of leadership attention to eCHIS, which was being overshadowed by competing priorities like the Community-Based Health Insurance (CBHI) program. One respondent highlighted, “*Though decentralization was made in the region, the leadership hasn*’*t engaged with eCHIS. They haven*’*t prioritized it like CBHI, and without their engagement, it's difficult to push the implementation forward”*. On a related note, a health worker pointed out, “*Leaders and mass media have given minimal attention to eCHIS compared to other initiatives like CBHI despite eCHIS being a major initiative”*. The strategy aims to alert and orient leaders to advocate for eCHIS, ensuring it becomes a priority at the kebele level.

#### 3.3.2 Develop clear guidelines and population data integration

Confusion regarding the appropriate population data source (FMOH vs. eCHIS) for planning and performance evaluation was a significant barrier. A respondent stated, “*We are still using a conversion factor based on population data that was enumerated 15 years ago for planning and performance evaluation. It leads to biased outcomes.”* Furthermore, one respondent added, “*In one kebele, the maternal and child health performance rate based on FMOH population data was calculated to be no more than 40%, but this figure could rise to 80% if eCHIS population data were used”* This strategy improves planning processes and builds confidence among health workers by developing formal implementation blueprints that incorporate local data.

#### 3.3.3 Enhance technical support and training programs

Inadequate post-training technical support and underutilization of devices, like tablets, were major barriers. One health worker noted, “*The system becomes unresponsive during data recording due to internet outages, disrupting client data and referral processes”* Additionally, the strong support from Health Information Technicians (HITs) was appreciated by interviewees, with one respondent mentioning, “*Whenever we face challenges with eCHIS, we can count on the HITs for assistance”* This strategy emphases on continuous training, mentorship, and technical support, ensuring system functionality and enhancing health workers' confidence in using eCHIS effectively.

#### 3.3.4 Foster community-level engagement and ownership

Low community awareness and insignificant engagement from structures like Women Development Groups were barriers to eCHIS adoption. A respondent point out, “*Only midwives and we are actively engaged in and concerned about the implementation of eCHIS”* Conversely, trust in health workers presents an opportunity for improving community engagement, as highlighted by a different health worker who mentioned the importance of effective communication networks, such as Women Development Groups. The strategy aims to involve community members to foster a sense of ownership, supporting maternal health tracking and referrals, thereby broadening participation and promoting the use of eCHIS at the community level.

#### 3.3.5 Create system interoperability and improve reporting capabilities

This strategy aims to address the issue of limited interoperability between eCHIS and the national DHIS2. One respondent explained, “*Although eCHIS includes a reporting feature, we continue to rely on manual methods for reporting HMIS indicators as the system's reporting capabilities are not yet mature enough”* In addition, another health worker highlighted the importance of system integration by stating, “*Ensuring interoperability between the eCHIS and DHIS2 systems is essential for establishing an effective reporting system”* By improving interoperability and automating reporting, this strategy reduces manual data entry and ensures reporting of data with high quality for decision-making.

#### 3.3.6 Establish a robust feedback and performance monitoring system

This strategy emphases on creating clear performance targets and structured feedback mechanisms to guide health workers in using eCHIS. One respondent noted, “*We don*’*t know how much we should reduce dropout rates or what completion rates we’re aiming for”* Another perspective came from a health worker who mentioned, “*There is a time when we received feedback from upper which was* generated from dashboard. *Though the feedback doesn*’*t come often, it helps us see where we are*”. This strategy would improve accountability, motivate health workers, and foster continuous quality improvement in service delivery by establishing formal feedback systems and utilizing the available dashboard data,

#### 3.3.7 Integrate eCHIS into health worker incentive programs

This strategy aims to address the lack of formal incentives/rewards for health workers using eCHIS, which has led to demotivation. One respondent shared, “*It's upsetting that despite the extra effort required to adopt eCHIS, there are no special rewards even recognition for those of us who have been early adopters”* Furthermore, a different worker pointed out similar frustrations, “*The lack of these incentives has led to bad feeling of demotivation among staff”* Introducing non-financial incentives, such as certificates of recognition, would boost engagement and compliance with eCHIS. This strategy promotes a culture of recognition and ownership. It also fosters long-term system adoption and improved performance (Table 2).

**Table 2:** Proposed eCHIS program implementation strategies aligned with the CFIR-ERIC framework.

Implementation strategy—ERIC strategy	Barriers to implementation	Facilitators to implementation	Mechanisms of action
(1) Strengthen Leadership Engagement and Advocacy - ERIC: Recruit, designate, and train for leadership	• Lack of leadership engagement at the kebele level. • Competing priorities, such as focus on the Community-Based Health Insurance (CBHI) program. • Lack of advocacy and awareness efforts	• Credibility and trust due to alignment with the Ministry of Health's Information Revolution agenda	- Increases leadership ensuring eCHIS is seen as apriority. - Leaders act as champions, securing resources, and ensuring that eCHIS receives attention and support at all levels
(2) Develop Clear Guidelines and Population Data Integration- ERIC: Develop a formal implementation blueprint	• Confusion around which population data to use (FMOH vs. eCHIS data) • Lack of standardized guidelines on integrating eCHIS data into routine planning	• Recognition of the importance of accurate, community-based data. • Government commitment to improve Health data management	- Enhances accuracy in planning, performance evaluation and reporting by integrating local population data. - Increases worker confidence by providing clarity on data use
(3) Enhance Technical Support and Training Programs- ERIC: Conduct ongoing training	• Lack of follow-up training or technical support after initial training. • Some devices (tablets) underutilized due to technical challenges	• Availability of Strong technical support. • Existing of Telegram plat forms	- Provides continuous learning opportunities through refresher training and mentorship. - Boosts confidence and technical skills. - Ensures a support system is in place to resolve technical and operational challenges
(4) Foster Community-Level Engagement and Ownership- ERIC: Involve patients/consumers and family members	• Low community awareness. • limited engagement of Women Development Groups	• Community trust in health workers; • effective communication structures like the Women Development Groups and One-to-Five networks	- Builds community ownership and ensures broader support for eCHIS, - enhancing participation in maternal health tracking and referrals
(5) create System Interoperability and improve Reporting Capabilities- ERIC: Develop and organize quality monitoring systems	• eCHIS reporting features are not fully developed. • Lack of interoperability between eCHIS and DHIS2. • Continued reliance on manual systems for reporting	• Support from development partners (for system improvements. • Existing demand for better data exchange and reporting functionality	- Integration of eCHIS with DHIS2 - Reduces data entry burdens by improving automated reporting features. - Provides timely and accurate data for decision-making and Health policy planning
(6) Establish a Robust Feedback and Performance Monitoring System-ERIC: Audit and provide feedback	• Absence of clear and measurable targets. • Lack of consistent performance Evaluation. • Limited use of dashboards	• Availability of dashboard data within eCHIS. • Existing informal feedback channels (e.g., via Telegram)	- Provides clear performance goals, improving accountability and motivation. - Encourages continuous quality improvement. - Uses dashboard data to inform decision-making
(7) Integrate eCHIS into Health Worker Incentive Programs-ERIC: Alter incentive/allowance structures	• Lack of formal incentive structures (non-financial). • Demotivation due to Absence of Recognition. • Competing priorities and focus on other programs (e.g., CBHI)	• Potential for introducing non-financial incentives (e.g., certificates of recognition, career advancement). • Willingness among health workers to adopt the system if properly incentivized	- Increases engagement and compliance with eCHIS. - Enhances motivation and ownership among health workers. - Creates a culture of recognition and reward, which promotes long-term system sustainability

## 4 Discussion

This study aimed to explore the barriers and facilitators affecting the implementation of the electronic Community Health Information System (eCHIS) and to formulate tailored implementation strategies. The implementation of the eCHIS reflects a complex interplay of facilitators and barriers across the five domains of consolidated framework for implementation research (CFIR) (25). Facilitators include its alignment with national health priorities, belief that eCHIS improves service delivery and data quality, and the system's adaptability and ease of use, which have promoted engagement among health workers. Similar studies conducted in Ethiopia also showed that electronic community health information systems (eCHIS) enhance data quality, usability, and service delivery for frontline health workers, primarily due to their adaptability and user-friendly design (23, 30). However, several barriers have limited its full impact, including unreliable internet connectivity, underdeveloped reporting functions, lack of clear implementation guidelines, and insufficient engagement from leadership Different studies conducted in other African countries including Ethiopia, Asia and South Americans also highlighted that the implementation of mHealth innovations has been hindered by the absence of clear implementation guidelines, limited leadership engagement and the lack of standardized operational processes (23, 31, 32). The findings highlight that for mHealth innovations such as eCHIS to be effective and sustainable over time, they require more than just technical robustness and alignment with national health priorities. They also need to be supported by strong governance systems, reliable infrastructure, and well-equipped health workforce.

To address these challenges, tailored implementation strategies are required, such as enhancing leadership engagement and advocacy, developing comprehensive guidelines, strengthening technical support, increasing community engagement, and improving system interoperability. These actions are crucial for ensuring the long-term sustainability and effectiveness of eCHIS in improving maternal health service delivery.

Leadership engagement emerged as a critical factor for the successful implementation strategy of eCHIS. Engagement of leaders signifies the active involvement of key decision-makers, including health administrators and local authorities, in prioritizing, advocating for, and allocating resources toward the system's implementation. The lack of attention from leadership, as highlighted by respondents, underscores the need for greater advocacy. This observation is consistent with the broader literature, which emphasizes that leadership involvement is vital for the adoption and success of mHealth initiatives (33). Studies in Ethiopia have shown that leadership commitment from the Ministry of Health played a pivotal role in advancing eCHIS (34). Similarly, mHealth interventions in Ghana have demonstrated the importance of leadership in ensuring that health systems receive the necessary support and resources (35). The alignment of eCHIS with the Ministry's Information Revolution agenda further strengthens the argument for prioritizing leadership advocacy to ensure eCHIS's success.

Insights from the interviews suggest that the development of clear guidelines and the integration of accurate population data are essential strategies for facilitating the implementation and long-term sustainability of the eCHIS. Integration of up-to-date and accurate population data would enable more precise planning and evaluation, leading to more reliable health outcomes and performance assessments. In turn, these elements would enhance trust in the system and promote its long-term adoption and sustainability, as health workers would be more confident in its utility and decision-making would be data-driven.

The confusion regarding population data sources used in planning and performance evaluation represents a significant barrier to eCHIS. One of the respondents highlighted that maternal and child health performance rates could improve significantly from 40% to 80% if eCHIS population data were used instead of Federal Ministry of Health population data. This issue resonates with findings from other mHealth interventions, where outdated or inconsistent data sources have resulted in biased outcomes (36). For example, in Afghanistan, mHealth interventions improved maternal and child health outcomes by integrating real-time local data (37). By developing clear guidelines that incorporate accurate population data, the implementation of eCHIS can improve planning accuracy and build trust among health workers, a strategy that has proven successful in other settings.

The provision of technical support and the implementation of continuous training programs was another strategy suggested by participants as critical components for enhancing the functionality of eCHIS. These factors are essential in ensuring that health workers have the necessary skills and resources to effectively use the system, address challenges, and maintain system performance over time. Continuous training ensures that users remain proficient in system operations, while ongoing technical support provides the needed assistance for troubleshooting and resolving technical issues, thereby improving overall system reliability and user confidence.

The need for ongoing post-training technical support was repeatedly emphasized by respondents, particularly in relation to internet outages and device malfunctions. Similar barriers have been documented in other studies. In Nigeria, public health nurses reported that consistent technical assistance was essential for the smooth integration of mobile technologies into their workflows (38). The presence of Health Information Technicians (HITs) who provide ongoing support was seen as a facilitator of eCHIS adoption, mirroring findings from other mHealth implementations where mentorship and technical assistance were crucial for success (39).

The findings underscore the significance of community engagement in influencing eCHIS adoption. Involving the community in the implementation process fosters a sense of ownership and support, which is vital for the system's acceptance and sustained use. Effective community involvement helps enroll mothers early into maternal health services and ensures continuity of care throughout pregnancy and postpartum periods. Engaging community members and leveraging local structures, such as women's development groups, enhances awareness and participation, which increases the utilization of eCHIS for maternal health services. Respondents noted that low awareness and involvement from community structures such as Women Development Groups limited the broader use of eCHIS. This finding is supported by research conducted in Madagascar, where community involvement and strong partnerships with local structures were instrumental in the successful implementation of a mobile health wallet for pregnancy-related healthcare (40). Studies from Ghana also suggest that trust in health workers and their use of mobile tools were important for fostering community engagement (35). Strengthening community-level ownership of eCHIS could increase its adoption and improve maternal health tracking and referrals.

Respondents emphasized the value of enhancing system interoperability and optimizing reporting capabilities in successful implementation of eCHIS. DHIS2 is the nationally adopted digital reporting platform within Ethiopia's health sector. eCHIS, on the other hand, is being implemented to support the digital provision of health services at the community level. To ensure efficient data management, the reporting modules of eCHIS need to be interoperable with the DHIS2 system. This would allow health extension workers to generate reports within eCHIS and electronically transfer them into DHIS2. Such integration would enhance reporting efficiency, minimize duplication, and improve the accuracy and timeliness of health data across both platforms. This improvement facilitates timely and informed decision-making in health management. Addressing this aspect enhances data accuracy, streamlines workflows, and optimizes the overall functionality of health information systems, ultimately leading to more efficient and effective healthcare delivery.

Interviewees suggested the continued reliance on manual methods for reporting health management information system (HMIS) indicators, despite the existence of eCHIS's reporting feature. This aligns with literature that highlights interoperability challenges as a common barrier in mHealth systems (33). In Brazil, similar issues were noted where mobile health systems faced integration challenges, resulting in redundant tasks for health workers (41). Addressing these challenges by improving system interoperability could reduce the burden of manual data entry and enhance reporting efficiency. Respondents highlighted establishing a robust feedback and performance monitoring system were essential strategies for motivating health workers and improving accountability in eCHIS implementation. These mechanisms provide health workers with clear performance metrics and regular feedback, helping them understand their progress and areas for improvement. By fostering a culture of continuous evaluation and guidance, these strategies not only motivate health workers but also ensure that they remain accountable to the goals of eCHIS, leading to better system performance and service delivery. Respondents noted the absence of clear performance targets, which hindered their ability to track progress. Evidence from Ethiopia and Afghanistan supports the role of regular feedback in improving health worker performance and fostering accountability (32, 36). Implementing formal feedback systems within eCHIS could provide health workers with the necessary guidance to achieve better outcomes and ensure continuous quality improvement in service delivery.

Strengthening internet connectivity and IT infrastructure was another strategy proposed for the successful implementation of eCHIS. This strategy aims to improve the technological foundation necessary for the efficient operation of eCHIS. This includes ensuring reliable, high-speed internet access and robust hardware and software systems, particularly in rural or underserved areas. Enhanced connectivity and infrastructure are essential for real-time data entry, synchronization, and reporting, which are critical for the smooth functioning of eCHIS. Participants reported frequent internet outages, which disrupted data synchronization and referral systems. This finding is consistent with global mHealth literature, where infrastructure and connectivity issues are frequently cited as barriers to successful implementation (26, 39, 44). In Nigeria, improving network reliability was considered essential for the sustainability of mHealth programs (38). Collaborating with Ethio-telecom to strengthen internet connectivity, particularly in underserved areas, would significantly enhance the functionality of eCHIS and reduce service delivery delays.

Finally, incorporating eCHIS into health worker incentive programs is regarded as a crucial strategy for enhancing the implementation of eCHIS. This approach recognizes that offering incentives, whether financial or non-financial, can significantly enhance the motivation and engagement of health workers. By aligning their efforts with tangible rewards or recognition, it promotes greater compliance with eCHIS processes, encourages sustained use of the system, and fosters a sense of ownership. Study participants expressed frustration that despite their efforts to adopt the system, they received no recognition or rewards. This aligns with findings from Madagascar, where an incentive program introduced to encourage the use of mobile health technology increased both participation and motivation (40). In Ethiopia, recognition and support from supervisors have been shown to facilitate the adoption of eCHIS (42). Integrating incentives, even non-financial ones such as certificates of recognition, could foster a culture of ownership and compliance among health workers, ultimately promoting the long-term adoption of eCHIS.

### 4.1 Strengths and limitations

The majority of existing studies have primarily focused on identifying barriers and facilitators to eCHIS implementation e.g., (24, 36, 39, 42, 43). A unique contribution of our study is its emphasis on developing tailored implementation strategies for the successful deployment of the eCHIS program. To our knowledge, this is the first study to systematically integrate the CFIR framework with ERIC strategies to identify barriers and facilitators, as well as customized implementation strategies specific to the eCHIS program. Additionally, the use of data from multiple stakeholders, including Health Extension Workers (HEWs), midwives, Health Information Technicians (HITs), Primary Health Care Unit (PHCU) directors, and Woreda health officers, enhances the credibility and dependability of the findings. Future research could focus on adapting the proposed strategies to other districts, as they present promising potential for broader applicability.

The limitations of our study include the context-specific nature of the findings, which may limit their applicability to other regions or settings, and the purposive sampling method used to select key informants and conduct in-depth interviews, which could have introduced bias. Furthermore, the results may not be generalizable to communities not represented in the study, and they do not necessarily reflect the views of health workers who did not participate. Another limitation of this study is that coding was conducted by a single researcher, and formal inter-coder agreement was not assessed. Although this may introduce the potential for individual bias in code interpretation, several measures were taken to enhance analytical rigor. These included repeated transcript reviews, the use of a well-established framework (CFIR) to guide coding, regular peer consultations, and the maintenance of an audit trail to document coding decisions and support transparency.

## 5 Conclusion

The study identified several key facilitators for the implementation of eCHIS, including its comprehensive design with integrated job aids, the availability of tablets and accessories, strong health worker self-efficacy, belief in the system's benefits for service delivery, and access to technical support and feedback mechanisms. On the other hand, implementation was hindered by perceptions of the system as externally imposed, lack of interoperability with existing systems, unclear implementation guidelines, weak community engagement, data synchronization issues, and limited leadership involvement. In response, seven tailored implementation strategies were developed to guide future efforts aimed at scaling up and sustaining the eCHIS program such as improving leadership engagement, developing clear guidelines, fostering community involvement, enhancing system interoperability, establishing robust feedback mechanisms, integrating health worker incentive programs, and expanding technical support. Incorporation of these implementation strategies could strengthen implementation, enhance adoption, and promote long-term sustainability.

The findings of this study can be used by stakeholders involved in the eCHIS program to design interventions that address the key implementation challenges identified. The CFIR-ERIC framework offers a comprehensive approach to linking implementation strategies with identified barriers and facilitators, ensuring that interventions are evidence-based and contextually tailored. We encourage future researchers and practitioners to adopt the integration of the Consolidated Framework for Implementation Research (CFIR) with Expert Recommendations for Implementing Change (ERIC), as this approach enhances the likelihood of successful program adoption, sustainability, and impact by aligning critical implementation factors with proven strategies.

## Data Availability

The raw data supporting the conclusions of this article will be made available by the authors, without undue reservation.
